# Caregiving, Pain, and Dementia: Evaluating the Role of Coping and Adaptation

**DOI:** 10.1093/geroni/igaa057.2841

**Published:** 2020-12-16

**Authors:** Sheria Robinson-Lane, Xingyu Zhang

**Affiliations:** University of Michigan, Ann Arbor, Michigan, United States

## Abstract

Pain is a stressor that can negatively influence quality of life for caregivers. Dementia caregivers have an increased risk for stress-related health outcomes including death. Few studies have examined the relationships between pain and coping-related outcomes for dementia caregivers. In the present study, Black family caregivers (n=56) completed a survey inclusive multiple health and coping measures. In addition to descriptive statistics, Pearson’s correlation coefficients were completed. 33% of caregivers experienced moderate to severe pain. The majority of participants with pain (72%) also had hypertension and were obese (69%). Pain intensity was significantly correlated with anxiety (p=.001). Effective coping and adaptation was correlated with perceived social support (p=.002) and perceived positive aspects of caregiving (p=.0.027). The primary coping strategies used by caregivers with chronic pain included spiritual coping, information gathering, reliance on past experiences, and maximizing resource use. Improving pain outcomes for caregivers may benefit both caregivers and persons with dementia.

